# CELDA – an ontology for the comprehensive representation of cells in complex systems

**DOI:** 10.1186/1471-2105-14-228

**Published:** 2013-07-17

**Authors:** Stefanie Seltmann, Harald Stachelscheid, Alexander Damaschun, Ludger Jansen, Fritz Lekschas, Jean-Fred Fontaine, Throng Nghia Nguyen-Dobinsky, Ulf Leser, Andreas Kurtz

**Affiliations:** 1 Charité-Universitätsmedizin Berlin, Berlin Brandenburg Center for Regenerative Therapies (BCRT), Berlin, Germany; 2 Institute of Philosophy, University of Rostock, Rostock, Germany; 3 Charité-Universitätsmedizin Berlin, Medical Informatics and Bioinformatics, Berlin, Germany; 4 College of Veterinary Medicine and Research Institute for Veterinary Science, Seoul National University, Seoul, Republic of Korea; 5 Humboldt Universität zu Berlin, Wissensmanagement in der Bioinformatik, Institut für Informatik, Berlin, Germany; 6 Max Delbrück Center for Molecular Medicine, Berlin, Germany

## Abstract

**Background:**

The need for detailed description and modeling of cells drives the continuous generation of large and diverse datasets. Unfortunately, there exists no systematic and comprehensive way to organize these datasets and their information. CELDA (Cell: Expression, Localization, Development, Anatomy) is a novel ontology for the association of primary experimental data and derived knowledge to various types of cells of organisms.

**Results:**

CELDA is a structure that can help to categorize cell types based on species, anatomical localization, subcellular structures, developmental stages and origin. It targets cells *in vitro* as well as *in vivo*. Instead of developing a novel ontology from scratch, we carefully designed CELDA in such a way that existing ontologies were integrated as much as possible, and only minimal extensions were performed to cover those classes and areas not present in any existing model. Currently, ten existing ontologies and models are linked to CELDA through the top-level ontology BioTop. Together with 15.439 newly created classes, CELDA contains more than 196.000 classes and 233.670 relationship axioms. CELDA is primarily used as a representational framework for modeling, analyzing and comparing cells within and across species in CellFinder, a web based data repository on cells (http://cellfinder.org).

**Conclusions:**

CELDA can semantically link diverse types of information about cell types. It has been integrated within the research platform CellFinder, where it exemplarily relates cell types from liver and kidney during development on the one hand and anatomical locations in humans on the other, integrating information on all spatial and temporal stages. CELDA is available from the CellFinder website: http://cellfinder.org/about/ontology.

## Background

**Figure 1:**
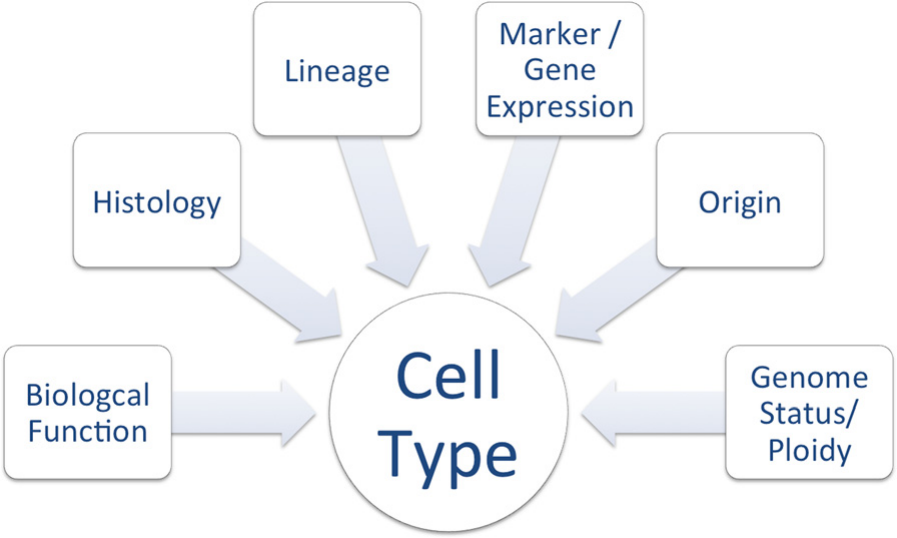
An overview about the different classes that are used to describe a cell type.

Cells are the building blocks of tissues and organs. The central importance of cells in the establishment and maintenance, as well as de- and regeneration of tissues, has long been recognized [1-3]. Intense research has led to the accumulation of an enormous and rapidly growing body of cell-related data in literature and different databases. These diverse datasets capture different dimensions of cells, including subcellular structures, transcriptome, temporal aspects such as the developmental stage or potency and spatial aspects like anatomical localization *in vivo* or cultivation conditions *in vitro* (Figure 1).

In order to compare, characterize and determine the type of cells based on cell-associated data, a systematic representation of cells is necessary. In particular, the context in which cell-associated data has been obtained must be properly represented, as differences may lead to grossly divergent characteristics and cell types. One prominent example for this fact is that stem cell differentiation *in vitro* frequently results in cells which have no known equivalence *in vivo*[4].

Several attempts have been made to present available information on cells in a unified format. For example the CELLPEDIA database [5] provides a richly annotated, intuitive resource for differentiated human cells by including numerous data types and relating these to tissues and organs in a way that makes it possible to directly compare different cell types.

Other data sources for cells include registry-like lists of cell lines and cell types such as the human embryonic stem cell database (hESCreg, [6]), the Characterization Tool [4] or StemDB [7] and cell line banks like the American Type Culture Collection (ATCC, [8]) or the European Collection of Cell Cultures (ECACC, [9]).

Furthermore, to comprehend the domain of cell types, several ontologies have been developed like the Cell Ontology (CL, [10]) and the Cell Line Ontology (CLO, [11]). Other ontologies describe the anatomy of organisms, which also include cells like the Foundational Model of Anatomy (FMA, [12]), the Human Developmental Anatomy (EHDAA, [13]) and the Mouse Adult Gross Anatomy (MA, [14]).

In summary, several data sources provide means to describe some of the many dimensions of information on cells, but none provides a truly comprehensive framework to address all or most of the available information (see Table 1). In particular, an extensive overview about cells of different species or developmental time points cannot currently be easily generated in these other databases.

**Table 1:** Resources of CELDA: This table lists elements that different sources of CELDA use to describe cell types (x = covered; o = partially covered)

	**Cells *in vivo***		**Cells *in vitro***		
	**CL**	**Cellpedia**	**CLO**	**hESCreg**	**Characterization tool**
**Biological function**	x				o
**Histology**	x	x		x	
**Karyotype**				x	
**Lineage**	x	x	x	x	x
**Marker**		x		x	x
**Nuclear Number**	x				
**Origin**	x	x	x	x	x
**Ploidy**	x	x		x	

Therefore, we developed a novel, comprehensive ontology, CELDA (Cell: Expression, Localization, Development, Anatomy) to formally represent important characteristics of cell types. CELDA is intended to structure the growing and diverse body of cell-related data. It allows for the description of cell types based on species, gender, anatomical location, subcellular structures, developmental origin and molecular composition such as gene expression. CELDA also enables the comparative description of the development of organs and tissues on the cellular level. Thereby, CELDA can be used to generate developmental trees of cell types, to compare cells *in vivo* with cells *in vitro,* or to find similar cell types in different organs, tissues or species. The ontology is designed to organize the vast and heterogeneous body of cell-related data by linking common elements through precise annotation and is adaptable to incorporate new information.

To establish CELDA, we addressed three fundamental issues: (1) Which data and data sources on cell type description are available? (2) Which properties are needed to fully describe cells *in vivo* and *in vitro*? and (3) How does an ontology need to be designed in order to allow a structured and standardized description of the properties defined in (2)?

## Methods

### Ontology development

Ontologies are formal explicit descriptions of general features of a certain domain. These general features are described within an ontology by stating necessary (or necessary and sufficient) membership conditions for classes (sometimes also called types). These classes are connected with each other by means of formal relations (which are also called object properties) [15].

In order to design an ontology development strategy, we consulted the guidelines by Noy and McGuinness [15], Bermejo [16] and Schulz et al. [17]. The first two are by now somewhat dated and also contended, but they were still useful to defined a four-step approach to develop CELDA:

1. Determination of the classes within the domain and scope of the ontology.

2. Evaluation of existing ontologies for their suitability of utilization.

3. Development of the fundamental outline of the ontology.

4. Formal representation of the ontology.

This four-step approach allows to create the ontology in a structured way. This general framework was combined with the modeling approaches suggested in the “Good Practice Ontology Design Principles” by Schulz et al., in particular for step 3 and 4.

To determine which classes are needed in the cell biology domain, we first analyzed existing ontologies and databases. In addition, the opinion of domain experts and scientists in the field of biology, medicine and cell biology were compared to identify criteria used to describe cell types. The distinction between cell types *in vivo* and *in vitro* happens to be crucial, as there is currently no data source that describes both variants of cell types comparatively and in detail.

We selected the Cell Ontology as the main source for cell types *in vivo* and the Cell Line Ontology, hESCreg, and the Characterization Tool as sources for cell types *in vitro*. Table 1 shows the properties used by these data sources to describe cell types, which formed the basis for selection of CELDA as shown in Figure 1. For describing both cell types *in vivo* and *in vitro*, we needed to cover biological functions (e.g. the barrier function), cytology and histology (including subcellular structures), lineage (using the relation develops-from), expressed genes, origin (e.g. anatomical location, species, gender, age) and the genome status (e.g. ploidy) of the cell types.

In order to decide which ontologies to integrate within CELDA, we performed an analysis of existing ontologies dealing with different aspects of describing cells. The Open Biomedical Ontologies Foundry (OBO) [18] provides a suite of orthogonal interoperable reference ontologies in the biomedical domain, including ontologies for cells, anatomy, molecular functions, cellular components, genes, proteins, phenotypes and development. Although none of these ontologies by themselves fulfill CELDA’s requirements, they provide a reasonable coverage of the domain if taken together.

Therefore, we decided to utilize as many classes as possible from these existing ontologies and to apply a top-level ontology which provides an ontological layer for linking and integrating various specific domain ontologies [19].

There are several upper-domain ontologies that have a focus on life sciences. We evaluated

Ontology of Biomedical Reality (OBR) [20]

Simple Bio Upper Ontology [21]

General Formal Ontology (GFO-BIO) [22]

BioTop [23].

We decided to use BioTop because it is founded upon formal design principles (as advocated by the OBO Foundry initiative), implemented in OWL2 and has a biological focus. Another advantage of BioTop is that it was actually designed to connect with important OBO Foundry ontologies and contains built-in bridge classes that serve this purpose [23].

BioTop can be used without an additional top-level ontology, but there are also bridges that allow its combination with either the Descriptive Ontology for Linguistic and Cognitive Engineering (DOLCE) [24] or the Basic Formal Ontology (BFO) [25]. BioTop itself is not part of the OBO Foundry [18], but the bridge allows it to import BFO, which is the top-level ontology that is part of and recommended by the OBO Foundry. We used relations as defined in BioTop and the OBO Relation Ontology (RO) [26].

In order to cover all the sub-domains needed for a full representation of all relevant aspects of cell types, we re-used existing ontologies and extended them when necessary. A list of the sub-domains can be seen in Figure 1.

Likely the most prominent ontology that describes cell types is CL, which describes *in vivo* cell types. It contains formal definitions for cell types, referring for example to the phenotypic characteristics of cell types [27]. CLO describes cell lines and their origins [11]. The Experimental Factor Ontology (EFO) [28] also contains a considerable number of classes for cell lines and cell types, linking cell line classes to both anatomical entities and diseases.

Biological processes are covered by both the Gene Ontology (GO) in its sub-ontology ‘Biological process’, as well as in EFO. The substructures of cells can be described by referring to the classes for subcellular structures in the GO [29]. Furthermore, some extensions and mapping ontologies are available from the OBO Foundry to extend the GO on behalf of cellular components and biological processes [30]. We also made use of these in order to develop a description of our domain as completely as possible.

The lineage of cell types is described in CL, CLO and EFO. These ontologies also partially address the origin of cell types, but only EFO contains terms to describe the species of origin. While both CLO and EFO contain terms to distinguish between sexes, only EFO contains terms for age. To fully describe the origin of cell types, ontologies from the anatomical domain can be used. The UBERON ontology [31] describes anatomical terms without reference to species, while other ontologies are specific to one species, like the Foundational Model of Anatomy (FMA) [12] and Human Developmental Anatomy (EHDAA) [13] for human or the Mouse Adult Gross Anatomy (MA) [14] for mouse. A mapping ontology from UBERON to species-specific ontologies like FMA or MA is also available at the OBO Foundry [32]. The genome status of cell types is partially described in the CL. A complete overview of the examined ontologies, their coverage of cell biological classes and further data sources are shown in Table 2.

**Table 2:** Classes for the representation of cell types and available ontologies and data sources which cover these domains

**Class**	**Information types**	**Ontologies**	**Other datasources**
**Cell types**	• Names and description of cell types	• CL [10]	
**Cell lines**	• Names and description of cell lines	• CLO [11]	• hESCReg [6]
		• EFO [28]	• Characterization Tool [4]
**Biological function**	• Biological function	• EFO	
	• Molecular processs	• GO [29]	
**Histology**	• Components and substructures	• CL	• hESCreg
	• Morphology	• GO	
		• GO_XP_ALL [30]	
		• Cellular_component_xp_self [30]	
		• Cellular_component_xp_go [30]	
		• Cellular_component_xp_cell [30]	
**Lineage**	• *In vitro*: origin cell	• CL	• hESCreg
	• *In vivo*: developmental tree	• CLO	
		• EFO	
**Marker/Gene Expression**	• Expressed genes	• Biological_process_xp_cell [30]	• Characterization Tool
		• Biological _process_xp_uber_anatomy [30]	• hESCreg
		• Biological_process_xp_multi_organism_process [30]	
		• Biological_process_xp_self [30]	
		• Biological_process_xp_cellular_component [30]	
**Origin**	• *In vivo*: anatomical location in tissue/organ	• CLO	• hESCreg
	• *In vitro*: environmental clue	• CL	
	• Species	• FMA [12]	
	• Gender	• MA [14]	
	• Age	• UBERON [31]	
	• Developmental stage	• EHDAA [13]	
		• uberon_anatomy_ontologies_bridge [32]	

The ontologies and bridges for mapping can be found at the OBO Foundry, http://www.obofoundry.org. hESCreg and the Characterization Tool are available at http://www.hescreg.eu and http://characterizationtool.cellnet.org.

For the construction of the ontology, we organized both the ontologies imported for re-use and the classes newly defined by us in a hierarchical structure using the top-level ontology BioTop together with the BioTop bridge to BFO and RO. This hierarchy is the backbone of CELDA. Since we intended to use CELDA as a basis for the CellFinder application (http://cellfinder.org), local copies of the ontologies were generated and imported into CELDA. This allows functionality of CELDA independently of external changes to some of the ontologies. When changes in one of the imported ontologies occur, CELDA can be tested with the new version of the ontology and after confirmation of stability, our local copy can be updated.

According to Courtot et al. [33], there are three general possibilities when referencing external ontology sources:

1. create own classes and reference other ontology classes,

2. generate and import modules of other ontologies,

3. import whole resources.

In most cases, we decided to import the whole resource. One major principle of the OBO Foundry is that every ontology should, in principle, cover a particular domain that is not covered by other ontologies. Hence, the ontologies we used from the OBO Foundry are intended to be orthogonal to each other and, thus, this approach did not lead to massive overlap. When no classes existed in the imported ontology for a cell type, cell line or anatomical entity we wanted to describe, we built the necessary classes ourselves and included them directly in CELDA. These classes were linked to classes from existing ontologies.

In a final step, we implemented the ontology in the Web Ontology Language (OWL, [34]). For this purpose, we used the ontology editor Protégé [35]. For automatic linking of classes from different ontologies, we used the Jena API [36], a Java framework for building semantic web applications. The Jena API was also used to add information from hESCreg and Characterization Tool databases to existing ontology classes and to develop new classes when necessary.

### Evaluation

Several approaches exist to evaluate new ontologies. Obrst et al. suggested six different evaluation techniques for the use of ontologies in life sciences [37-39]:

1. assessment by humans against a set of criteria

2. comparison of the ontology against a set of criteria

3. evaluate the use of the ontology in an application

4. natural language evaluation techniques

5. ontology accreditation and certification

6. use reality as a benchmark.

We created CELDA especially for use in CellFinder; therefore, our focus for evaluation was the performance of CELDA within this application (technique 3). In addition, we compared CELDA with existing ontologies and data sources during the whole developmental process (technique 2). The ontology was also checked by domain experts (technique 1).

## Results

### Developing the fundamental outline

**Figure 2:**
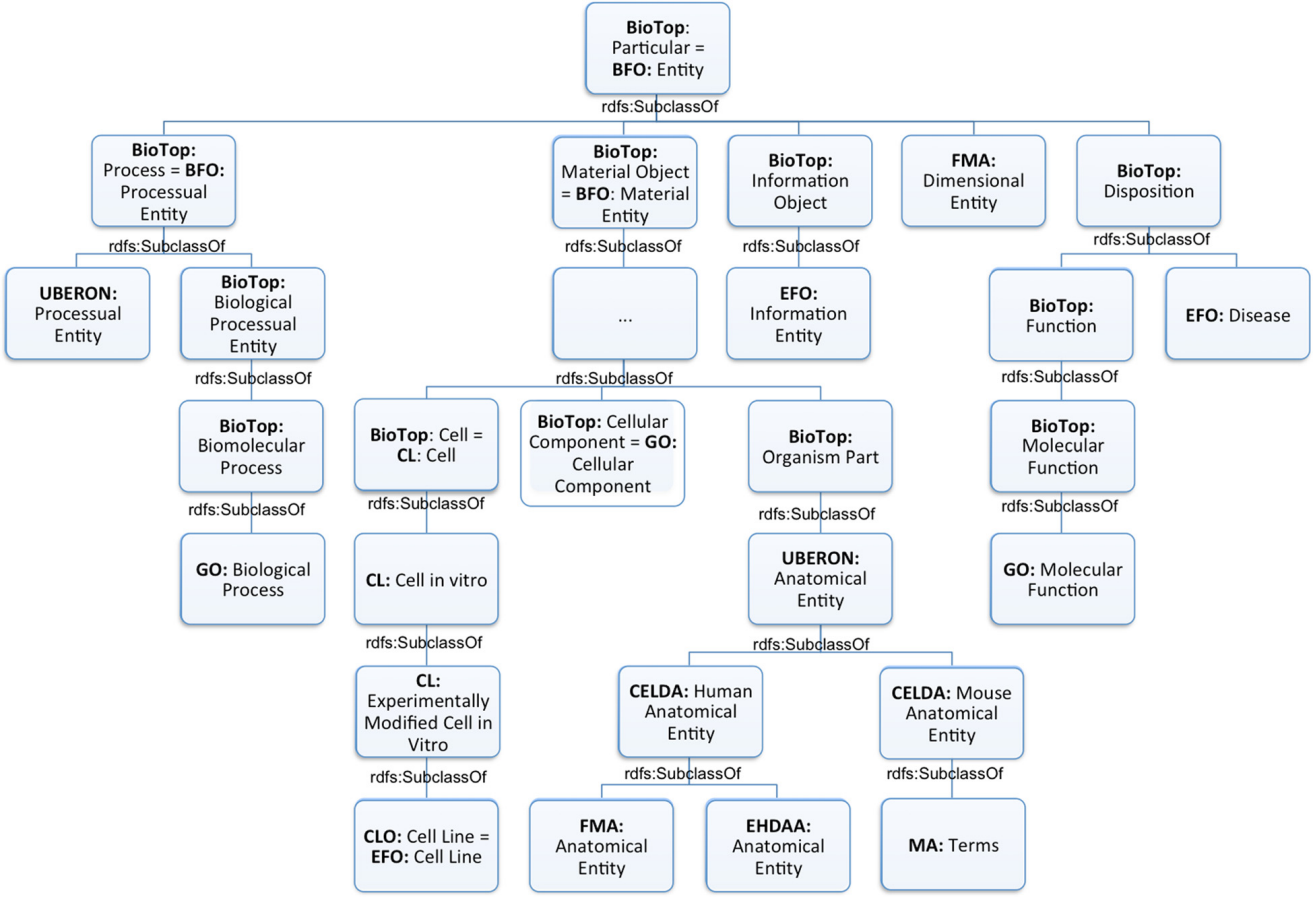
**Linking of ontologies in CELDA using BioTop.** This diagram shows how high-level classes of CL, CLO, EFO, EHDAA, FMA, GO, MA and UBERON are linked together in CELDA using BioTop. Read from top to bottom, the lines represent *rdfs:subclassOf* relations.

When analyzing the available ontologies describing one or more of the domains needed to describe cell types (see Figure 1), we found eight ontologies covering at least one of these domains: CL, CLO, EFO, EHDAA, FMA, GO, MA and UBERON. These were then linked using the top-level ontology BioTop as shown in Figure 2. CLO and EFO already provide a connection to BFO. Therefore, we also used a bridge between BioTop and the BFO and RO [40].

CL was linked to BioTop by defining *CL:Cell* as equivalent to *BioTop:Cell*. UBERON was linked to BioTop with a *rdfs:subclassOf* relation between *BioTop:organism part* and *UBERON:anatomical entity* and with a *rdfs:subclassOf* relation between *UBERON:processual entity* and *BioTop:Process*. To link the EHDAA, the FMA and the MA with BioTop, we created two new classes: *CELDA:human anatomical entity* and *CELDA:mouse anatomical entity*. These two classes were linked to *UBERON:anatomical entity* with a *rdfs:subclassOf* relation. We then linked *FMA:anatomical entity* and *EHDAA:anatomical entity* with *CELDA:human anatomical entity* and all top-level classes from the MA with *CELDA:mouse anatomical entity* using the *rdfs:subclassOf* relation. The three GO root classes were also linked to BioTop by the *rdfs:subclassOf* relation. We linked *GO:biological process* with *BioTop:bio molecular process* and *GO:molecular function* with *BioTop:molecular function.* Finally, we declared *GO:Cellular Component* to be equivalent to *BioTop:Cellular Component.*

After creating this scaffold for CELDA, we used the reasoner HermiT version 1.3.6. [41] to test the logical consistency after the linkage of the diverse ontologies. During the classification, no inconsistencies were reported by HermiT.

We then imported the entirety of resources from CL, CLO, EFO, EHDAA, FMA, GO, MA and UBERON into this scaffold. To make sure that the classes of the diverse ontologies were properly mapped to each other, we used ten mappings and bridges provided by the OBO Foundry (Table 1):

• cellular_component_xp_self.

• cellular_component_xp_go.

• cellular_component_xp_cell.

• Biological_process _xp_cell.

• biological _process_xp_uber_anatomy.

• Biological_process_xp_multi_organism_process.

• biological_process_xp_self.

• biological_process_xp_cellular_component.

• uberon_anatomy_ontologies_bridge [32].

• molecular_function_xp_uber_anatomy [42].

For some of the imported ontologies, no bridges were available. For these ontologies, we created mappings ourselves using the JENA API. New bridges were created between:

• CL and MA.

• CL and FMA.

• EHDAA and FMA.

• CL and UBERON.

Altogether, more than 196.000 classes for cell types, cell lines and anatomical entities were included from eight different ontologies in the CELDA ontology. In CELDA, 15.439 classes were newly defined together with 203.058 relations involving these classes. The OWL file CELDA_import.owl contains the top-level structure as well as the newly-defined classes together with the axioms stating the relations of these classes to classes from other ontologies. The file CELDA.owl imports CELDA_import.owl together with all ontologies and bridges as described above.

### Representation of cell types

**Figure 3:**
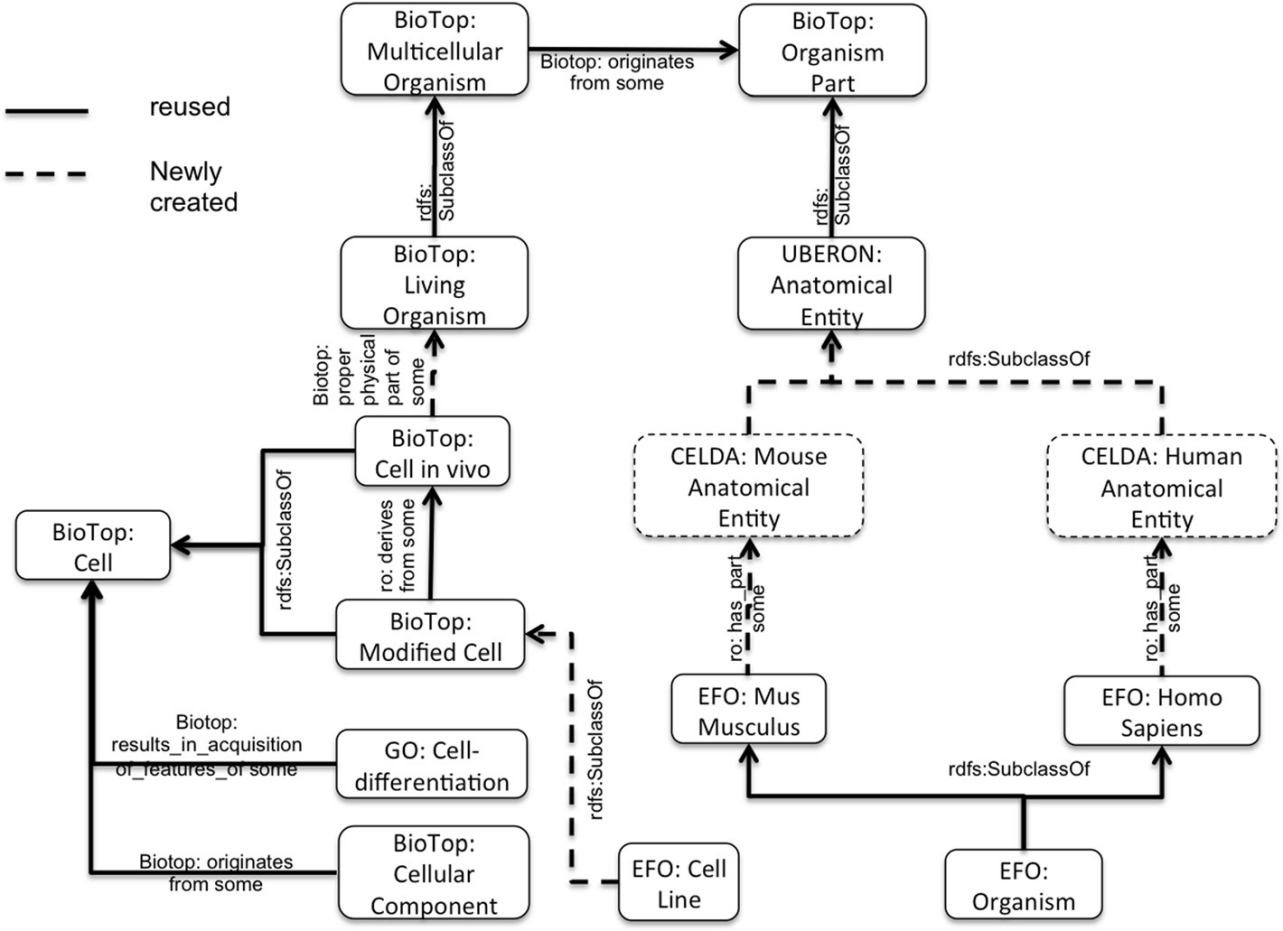
**Domain representation in CELDA.** CELDA distinguishes between cells and cell lines. Subclasses of cells describe groups of cells with similar properties like developmental stage, anatomical location, cellular structures or expressed molecules *in vitro* or *in vivo*. Cell lines are permanently established cell populations consisting of cells *in vitro* from exactly one cell type.

The structure shown in Figure 3 gives an overview of how we represent the domains needed to describe cells (see Figure 1) in CELDA with some of the high-level classes.

Among the generic names for cells, we distinguish names for cell types and cell lines. Cell type names are general terms that describe cells with similar or identical properties (e.g. “embryonic stem cell”, “induced pluripotent stem cell”), while names for cell lines refer to groups of similar cells of an *in vitro* type (e.g. “hESC line H1”, “lung fibroblast line IMR90”). Cell lines, on the other hand, are permanently established cell populations consisting of cells *in vitro* from exactly one cell type. Such a population can be modeled as a collection with *in vitro* cells as grains [43]. While cell types find their way into CELDA as subclasses of the class *Cell*, the grains of cell lines are instances of *CL: experimentally modified cell in vitro*.

There are important relations between cells such as the ability for one to develop from another cell. A cell *in vitro* can be derived from a cell *in vivo* of a certain type. Cells can be characterized by their location (within an anatomical entity), their subcellular structures and their expression patterns, i.e. by the biomolecules they contain. Such characterizations may also allow finding cell types with similar properties from different species. A cell can be an organism by itself (prokaryotic cells) or part of an organism. OWL would allow the inclusion of individuals in order to represent particular cells, though this would transcend the purpose of an ontology proper.

**Figure 4:**
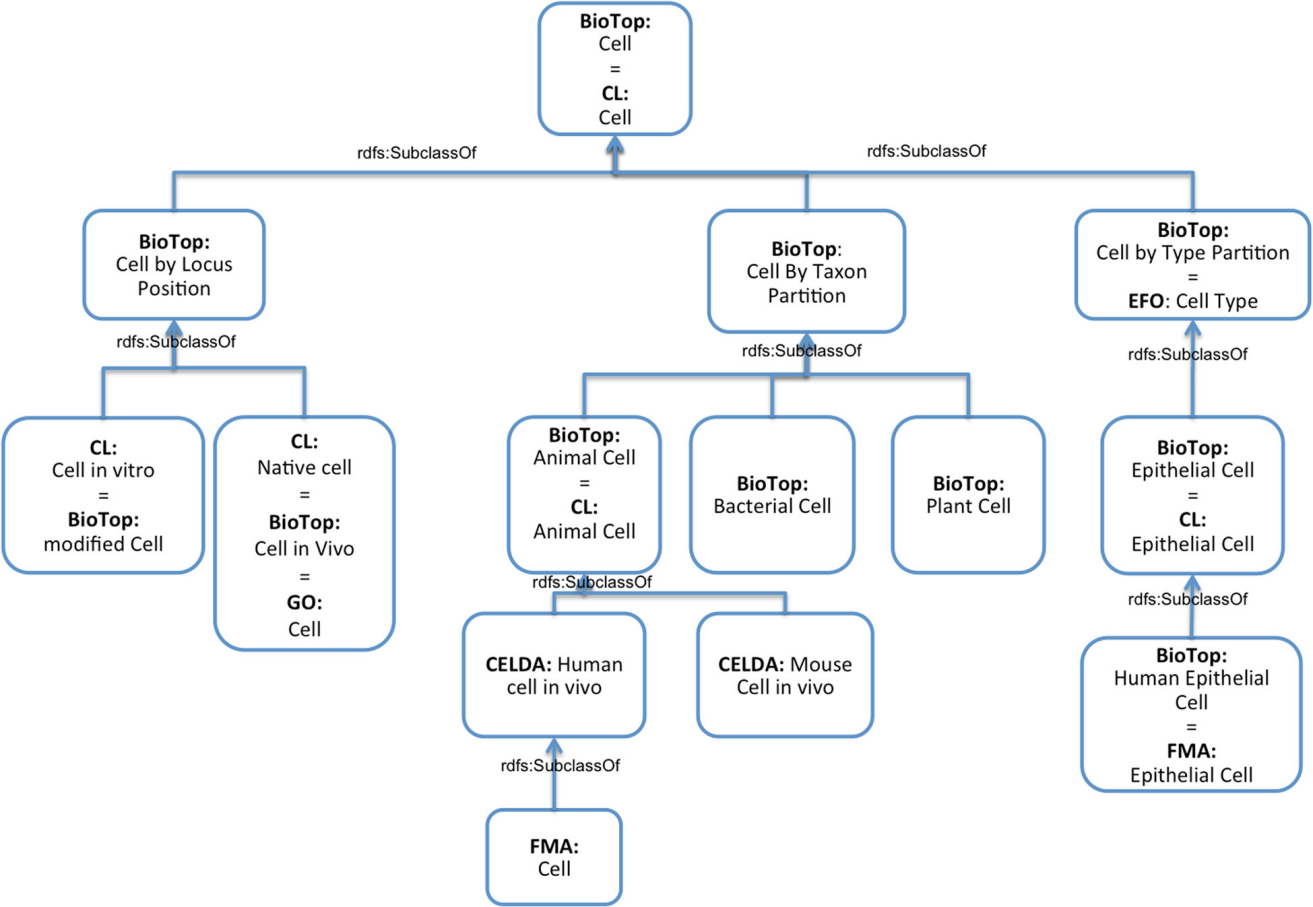
**How to connect various classes with the label “cell”.** Several of the ontologies imported into CELDA contain classes labeled “cell” or “cell type”. The figure shows which of these are stated to be equivalent or connected by means of *rdfs: subclassOf* relations in CELDA.

Most of the imported ontologies contain a class called “cell” or “cell type”. Therefore, careful analysis and the relation of these terms to each other was needed to define which terms are equivalent to each other and which classes are to be treated as subclasses. The result is shown in Figure 4. The EFO contains a class called “cell type” with a matching definition (“distinct morphological or functional form of cell”), but the subclasses (e.g. “blast cell” or “fibroblast”) make it clear that the instances of these classes are, in fact, cells and not cell types or cell forms. For this reason, we interpreted *EFO:cell type* as a class of cells whose subclasses categorize cells according to their type or form.

### Application of CELDA within the cell-related data repository CellFinder

**Figure 5:**
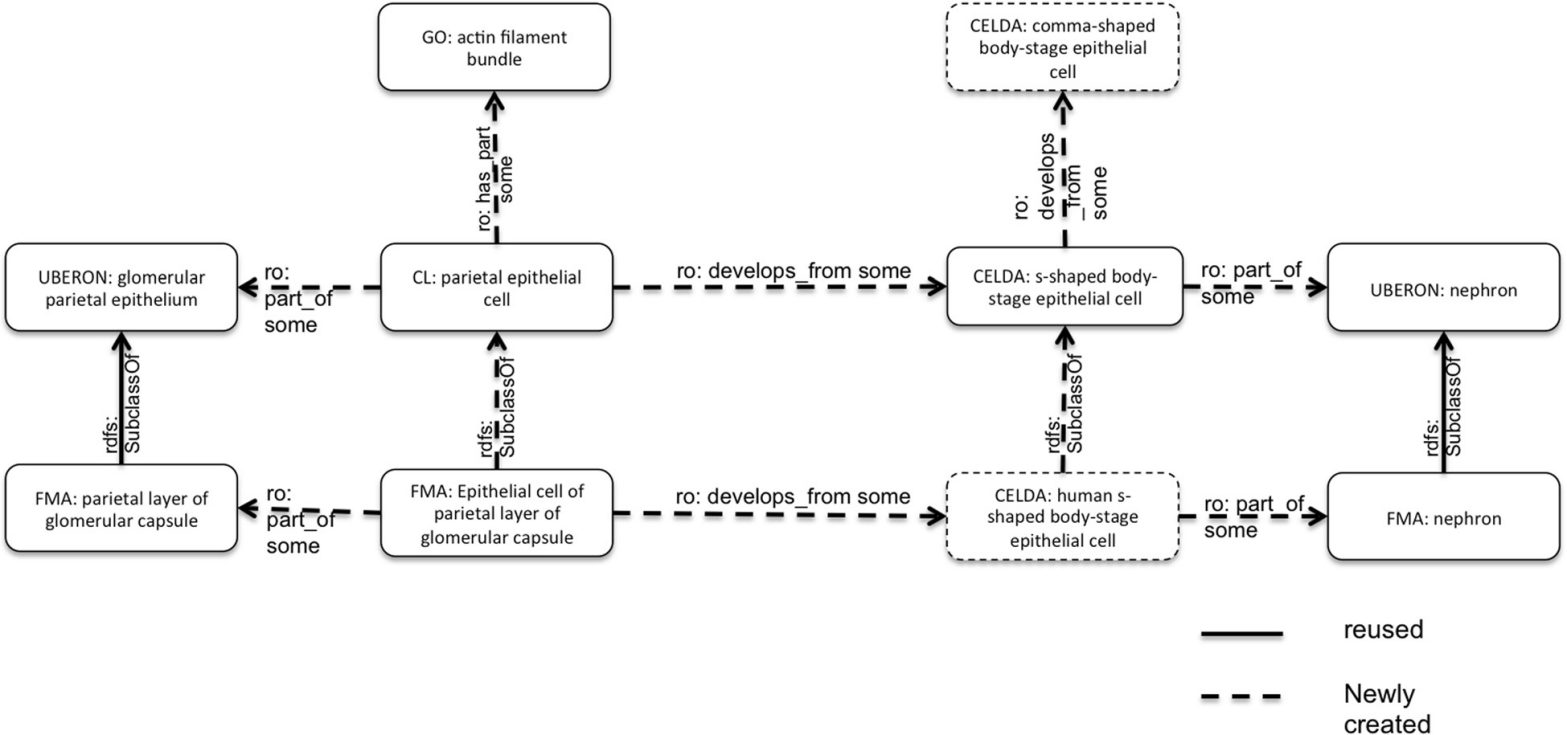
**An extract from the representation of a ‘parietal epithelial cell’ in the CELDA ontology.** Extra classes for a detailed description of these cell types were included, and the *ro:develops from* relations where added. Furthermore, we linked the cell types to the anatomical entities where they occurred.

CELDA has been developed in order to be used within the CellFinder project (http://cellfinder.org). CellFinder is a web-based data repository for scientific statements about cell types created and maintained at the Berlin-Brandenburg Centre for Regenerative Therapies (BCRT). CellFinder is aiming at an integral representation of data on cell types in the context of molecular, phenotypic, anatomical and developmental information in health and disease across species. CELDA is used as a backbone within CellFinder to enable integration of diverse primary datasets. An important application of CELDA is to link and organize *in vivo* and *in vitro* derived data regarding cell types (e.g. gene expression profiles, morphological and anatomical information including images), the origin of the cell types, references and the relations among these datas. For example, CELDA was used to position *human parietal visceral epithelial cell* in the developmental tree of kidney cell types and to find cell lines with similar descriptions (Figure 5). Furthermore, it is now possible to associate images of cells displaying morphologies, anatomical location or subcellular structures with gene expression data. These can be further related to the spatial and temporal context of the cell type. An example of the context of a cell type is the glomerulus and the kidney for human glomerular visceral epithelia. Any neighboring, similar or related cell types in other species can then be directly identified using a class from CELDA.

**Figure 6:**
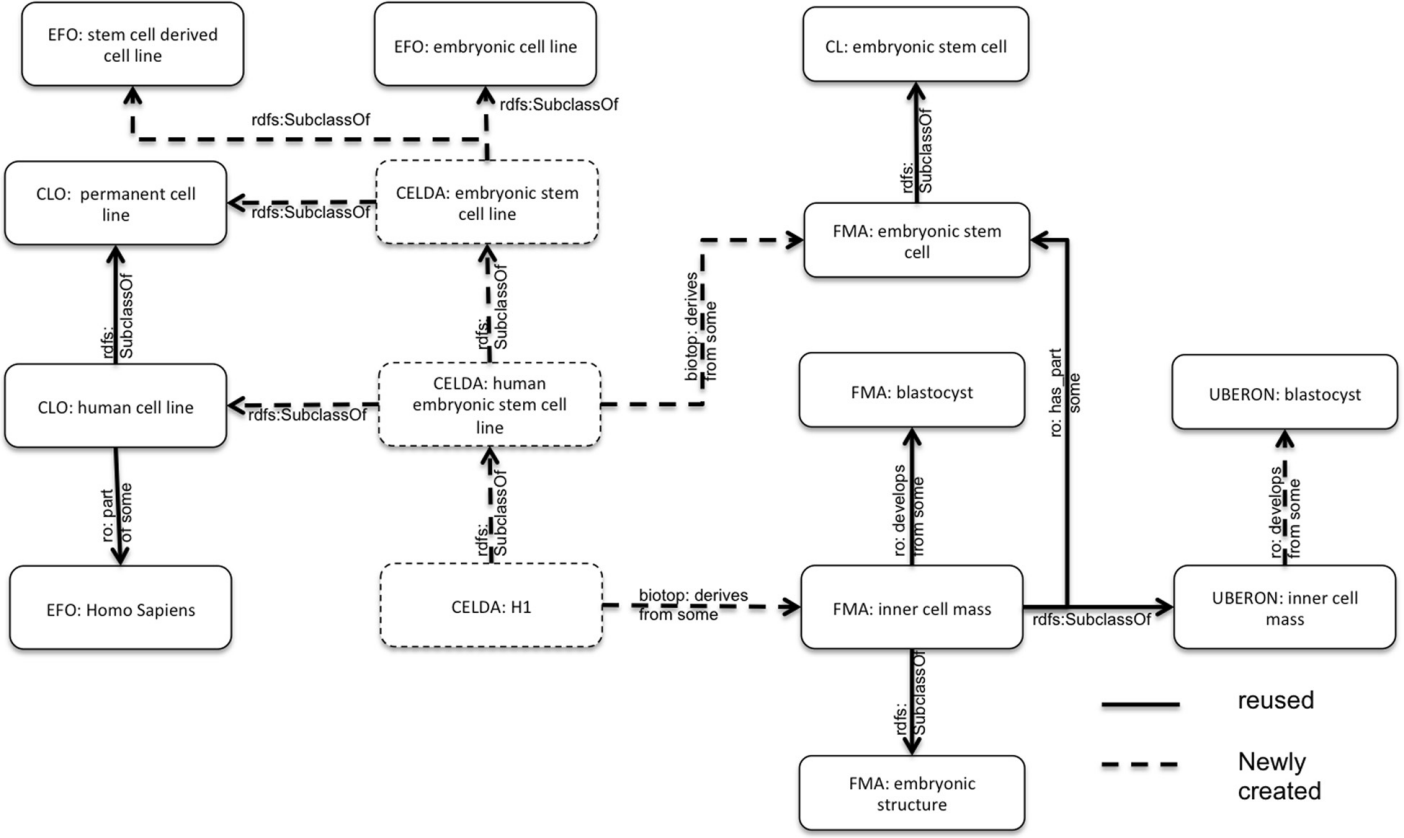
**An extract of the representation of the human embryonic cell line H1 and the relation to its *in vivo *counterpart in the CELDA ontology.** We added new cell lines like H1 as described in hESCreg when they are not already contained in the CLO.

CELDA also allows comparing *in vivo* cell types with cell lines *in vitro.* Figure 6 shows an example of a human embryonic stem cell line *H1in vitro* that is derived from the inner cell mass of a human embryo, which is part of the human blastocyst *in vivo*.

### Evaluating CELDA in the kidney domain

The degree and accuracy of characterization and analysis within CELDA depends largely on the cell-related data that is integrated in the ontology. To test the application of CELDA on the representation of developmental trees, we chose to represent cell types involved in human kidney development in order to reduce the data scope. Using a set of domain related publications and online tools [44-53], the developmental tree for human kidney cells starting from the zygote was created by domain experts working in the field of kidney regeneration. Altogether, we found 145 cell types taking part in kidney development. 75 of these cell types in the developmental tree were not part of the CL and were included in CELDA based on manual data extraction from the sources mentioned above. The developmental tree can be viewed online at http://cellfinder.org/development.

As the cell types described in CL are species-independent, we extended CELDA with species-specific classes for each of the 145 identified cell types relevant for kidney development when no equivalent class was found in FMA or EHDAA for human or in MA for mouse. These classes were linked to the species-independent classes using a *rdfs:SubclassOf* relation.

**Figure 7:**
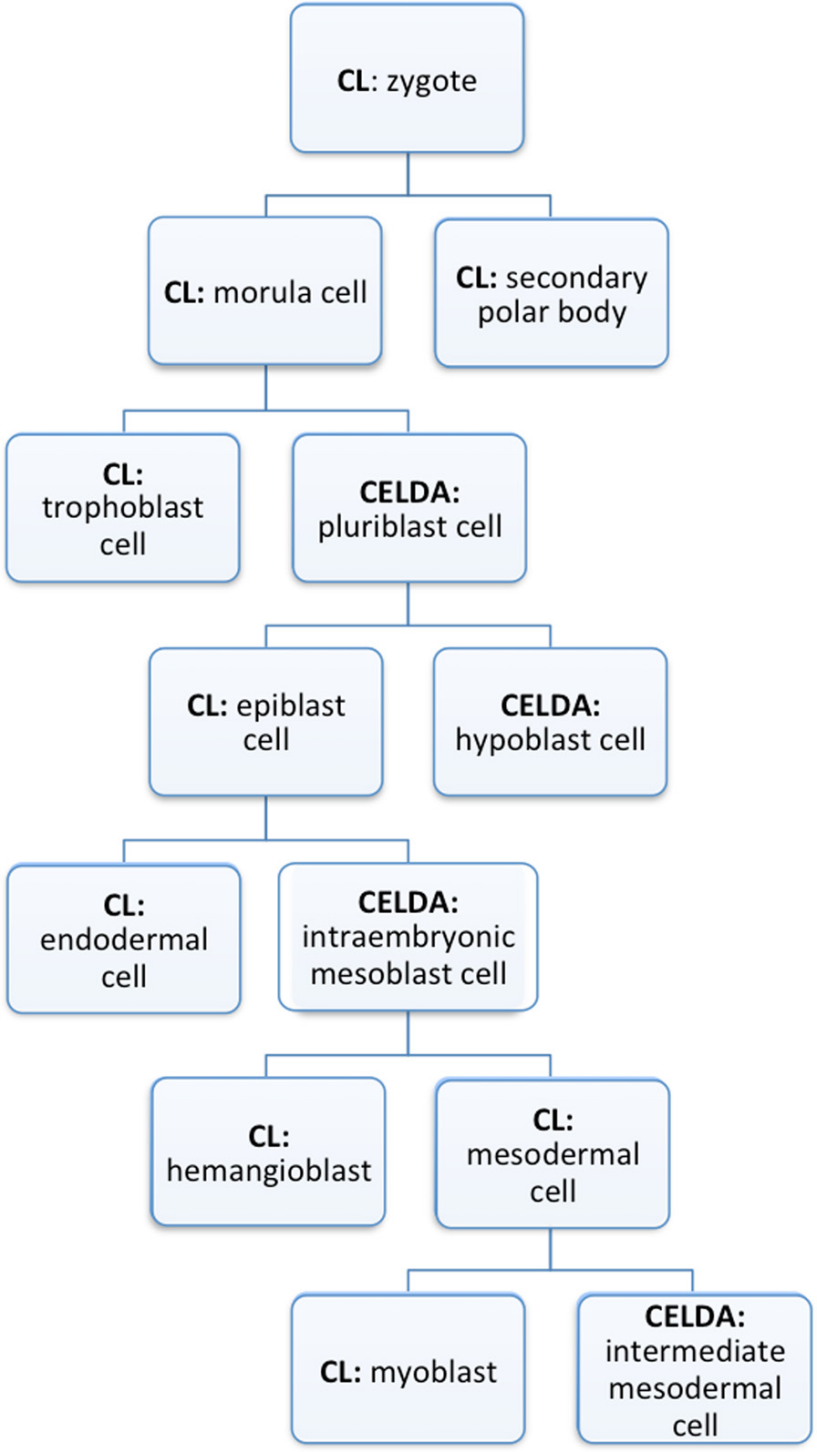
**An extract of the developmental tree of cells that is part of the CELDA - Ontology.** Read from top to bottom, the lines represent the *ro: develops from* relation.

These classes were then linked to UBERON for species-independent cell types using the *ro:part_of* relation. Human-specific cell types were linked to classes in FMA and EHDAA and mouse-specific cell types were linked to classes in MA. Furthermore, the types were linked among each other using the *ro:develops_from* relation to represent the developmental history of cells. A section of the developmental tree generated with CELDA, showing the development of a myoblast, can be seen in Figure 7.

When a cell type is characterized by specific cellular structures, this class is linked to classes from GO describing the cellular structure using the *BioTop:has_component_part* relation .

## Discussion

CELDA is an ontology for the formal description of cell types built by using and carefully extending existing ontologies from the biological domain. It integrates different dimensions of cell types at the genetic, molecular, structural, functional, temporal and spatial level. It also addresses the organization of cell types into higher-order structures (e.g. develops from or anatomical location).

CELDA aims to reflect the language used by researchers in laboratories and publications to distinguish between cell types. Accordingly, the design process of CELDA could be described as being data-driven because we focus on representing those kinds of data which are regularly generated in laboratories and on making them intuitively useable for semantic inferencing over cell types. A disadvantage of such a data-driven process in contrast to a model-driven process is the need for change when new technologies emerge. However, new technologies can be integrated into CELDA and would only require slight modifications. We believe that the ways in which cell types are described will most likely be straightforward extensions of our core model, especially when novel molecular, substructural and physical data become available.

When re-using existing ontologies, we store them on our server and use this version instead of using other versions provided, for example, by the OBO Foundry or by the authors of the ontologies. This allows CELDA to be independent of these data sources and assures that the ontologies are available in the version needed. This is important for the reliable functioning of any application that, like CellFinder, uses CELDA as its data structure. The disadvantage of this approach is that the ontologies are not always up-to-date; it is necessary to search manually for updates, check their compatibility and then integrate them.

CELDA was designed to allow researchers to find cell types through their properties and to reason about various kinds of relationships between different cell types. The availability of data from diverse domains makes it possible to access and find cells with limited data from different domains and relate them with similar cells, e.g. of different species, developmental stage or from the *in vitro* or *in vivo* domains (See Figure 6). By serving as a data model for the CellFinder data repository, CELDA has provided a proof of concept that it may be suitable for scientific applications in cell biology.

Up to now, CELDA primarily includes data on the development of the human kidney on a cellular level. The current work on CELDA focuses on describing the development of liver and skin cell types and to characterize stem cells *in vivo* and *in vitro* in developmental differentiation.

When creating CELDA, we faced several problems. One major problem was the occurrence of similar or identical classes in different ontologies. We tried to find them automatically by searching for identical names and using reasoning, but due to variant forms of spelling and the use of synonyms, it was not always possible to automatically find all occurrences. We are working on identifying equivalences manually, but the completion of this task will require some time due to the enormous and increasing amount of data. It will be of great benefit if further updates of the integrated ontologies deal with such problems like using a standardized naming convention or including references to equivalent classes in other ontologies.

Another problem occurred when dealing with terms that occur in the same or in similar forms in several of the integrated ontologies, perhaps with different explicit definitions. Major examples are, obviously, the terms “cell” and “cell type”. The label “cell”, e.g., could be used for the class of all cells, (in GO) for the class of cells *in vivo*, or even (in FMA) for the class of human cells *in vivo*. The label “cell type” probably rests on a confusion between the two relations of instantiation and subclasshood (which are not distinguished, e.g., in many thesauri [54]). While subclasses of the class *Cell* are, of course, types of cells, all instances of *Cell* are particular cells that exist at a certain time and could, e.g., be viewed under the microscope. A closer inspection of the use of the label “cell type” showed that its instances (or at least instances of subclasses) were, in fact, meant to be particular cells. The general lesson to be learnt is that ontology designers should be very careful with the naming and should also have an eye on problems that could occur when the ontology to be developed is combined with other resources. Naming conventions, like those provided by the OBO Foundry community [55], can help to prevent such problems.

A difficult issue was brought up by the question of when to call a cell a human cell. In canonical cases, human cells are (1) part of a living human organism and (2) derive from a human zygote. A baboon heart transplanted to a human organism may fulfill the first criterion, but not the second, whereas human brain cells transplanted to mice fulfill the second criterion, but after the transplantation no longer the first. Cells of multicellular organisms are called “*in vivo* cells” if they are part of a living organism. The existence of xenografts, however, shows that we have to distinguish between several cases: (1a) cells within the organism that developed from the same zygote from which the cell developed, (1b) cells within an organism that developed from a zygote of the same species as the zygote from which the cell developed, and (2) cells within an organism of a different species. The strictures of description logics (on which OWL is based) do not allow us to distinguish between the cases (1a) and (1b), but they allow us to set off (2) as a distinct possibility.

In CELDA, we decided to reserve the label “human cell *in vivo*” for cells only that fulfill both criteria, i.e. that developed from a human zygote and are part of a living human organism. This implies that a human cell which is transplanted to another organism does not count as “human cell *in vivo*” anymore. This decision was motivated by the biological point of view that we use to describe cells. From the biological point of view, xenografts are artificial systems and not in their natural environment. As the canonical environment of cells would be too complex to be described in detail, we need to reserve the modifier “*in vivo*” as a shortcut for those cells that interact with their canonical environment and are likely to show typical properties, whereas cells outside of this canonical environment may behave quite differently. In order to also cover the non-canonical cases, we included a class *Xenograft cell* in CELDA, which can be used to describe such transplanted cells.

Using an ontology to organize the information on cells provides the possibility for automatic reasoning to gain new hypotheses as described by Meehan et al. [27]. They expanded the CL and used automatic reasoning to find mistakes in the ontology. The reasoners exposed areas in the ontology where new classes were needed to accommodate species-specific expression of cellular markers and inferred new relationships within the CL and between the CL and the contributing ontologies. Furthermore, reasoners allow finding inconsistencies in the ontology as we have shown in the result section. Unfortunately, current reasoners have limitations when dealing with large ontologies. For example, HermiT [41] only supports the usage of 1 GB RAM. Thus, in the case of CELDA, reasoning over the complete ontology is problematic. Therefore, we decided to use reasoning only as a tool to check parts of the ontology for inconsistencies as detailed above in the result section. However, it would be very desirable to reason with the complete resource or at least with larger parts of CELDA in order to gain new hypotheses on cell function based on similarity to other cell types. We hope that future versions of reasoners, in combination with improved hardware, will be able to process large ontologies.

Finally, it should also be noted that CELDA, like many other ontologies, is not yet complete. Many developmental processes in organisms are, at present, only described for tissues, but not on the cellular level. Therefore, CELDA can grow when future research provides new insights. Given the large number of classes to be represented and the growing and still versatile body of knowledge in this domain, it is unrealistic to use manual curation as the sole strategy. However, the development of automatic mechanisms to limit or eliminate duplications and redundancies on the top level remains an ongoing task.

## Conclusions

The new ontology, CELDA, integrates and extends already existing ontologies in order to represent cell types as described in current research. CELDA relates cell types not only to other cell types, but also to anatomical components and cellular structures from other OBO library ontologies. In the long term, we expect that biological databases will move beyond a histology and gene-centric view and biological mechanisms will be studied at a more integrated level. Since cells are the biological units from which tissues, organs and phenotypes are built, this transition will be facilitated by rich and explicit description of cell types across phyla that can be adapted by biological databases. We believe that CELDA will support standardization and comparability of the complex datasets for each cell and organize these in a cellome environment. The outline of CELDA provides for the first time a solution to facilitate the integration of ontologies from multiple domains that can also be applied to other cell-centered data repositories.

## Competing interests

The authors declare that they have no competing interests.

## Authors’ contributions

AK, SS, TNND, UL, LJ and HS participated in the design of the ontology. SS implemented the model to represent CELDA in a RDBMS and FL, JFF and AD participated in testing it and contributed in the refinement of the work. UL and LJ participated in designing the ontology relationships and its logical outline. All participated in drafting and revising the manuscript. All authors read and approved the final manuscript.
